# The one-dimensional coordination polymer poly[tetra­kis­[(4-chloro­phen­yl)methanaminium] [cadmate-μ-cyclo­hexa­phospho­rato]]

**DOI:** 10.1107/S160053681104133X

**Published:** 2011-10-22

**Authors:** Sonia Abid, S. Salem Al-Deyab, Mohamed Rzaigui

**Affiliations:** a Laboratoire de Chimie des Matériaux, Faculté des Sciences de Bizerte, 7021 Zarzouna Bizerte, Tunisia; b Petrochemical Research Chair, College of Science, King Saud University, Riyadh, Saudi Arabia

## Abstract

Cyclo­hexa­phospho­ric acid (P_6_O_18_H_6_) reacts with cadmium carbonate and 4-chloro­benzyl­amine (CBA) to give the mononuclear title complex, (C_7_H_9_ClN)_4_[Cd(P_6_O_18_)]_*n*_, in which the Cd^II^ atom, lying on an inversion centre, has an octa­hedral coordination built of six O atoms of two centrosymmetric P_6_O_18_ rings. Each P_6_O_18_ ligand acts as a bridge, linking two Cd^II^ atoms and forming an anionic coordination polymer [Cd(P_6_O_18_)^4−^]_*n*_ extending along [010]. Adjacent polymeric chains are connected through N—H⋯O and C—H⋯O hydrogen bonds, generating a three-dimensional supra­molecular network.

## Related literature

For the crystal chemistry of condensed phosphates, see: Averbuch-Pouchot & Durif (1996 ▶); Durif (2005 ▶). For general background to supra­molecular complexes, see: Kolotuchin *et al.* (1995 ▶); Tong *et al.* (1999 ▶). For Cl⋯Cl inter­actions, see: Hathwar *et al.* (2010 ▶) and for π–π inter­actions, see: Janiak *et al.* (2000 ▶). For the synthesis, see: Schülke & Kayser (1985 ▶). For related structures, see: Du *et al.* (2010 ▶); Hu *et al.* (2008 ▶); Kontturi *et al.* (2005 ▶); Man *et al.* (2006 ▶).
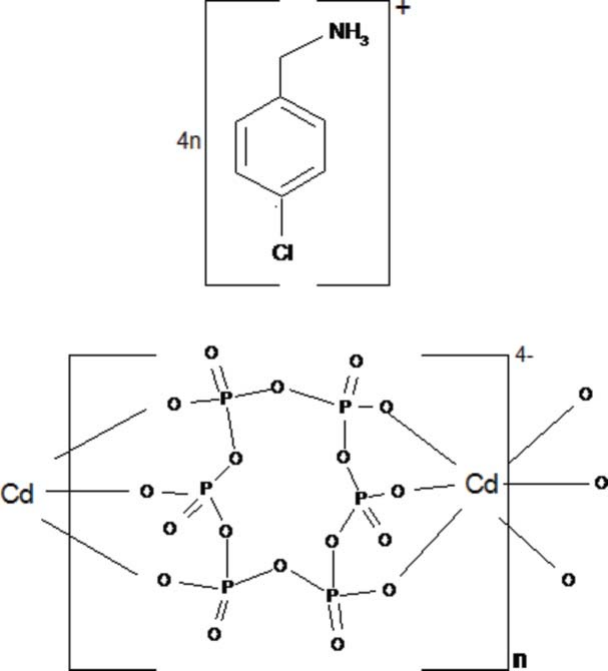


## Experimental

### 

#### Crystal data

(C_7_H_9_ClN)_4_[Cd(P_6_O_18_)]
*M* *_r_* = 1156.63
Triclinic, 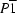

*a* = 8.021 (4) Å
*b* = 8.1696 (16) Å
*c* = 17.919 (3) Å
α = 87.31 (5)°
β = 88.914 (19)°
γ = 70.100 (3)°
*V* = 1102.9 (6) Å^3^
*Z* = 1
Mo *K*α radiation
μ = 1.03 mm^−1^
*T* = 293 K
0.22 × 0.20 × 0.18 mm

#### Data collection

Enraf–Nonius TurboCAD-4 diffractometer
3873 measured reflections
3770 independent reflections
3506 reflections with *I* > 2σ(*I*)
*R*_int_ = 0.009
2 standard reflections every 120 min  intensity decay: 1%

#### Refinement

*R*[*F* ^2^ > 2σ(*F* ^2^)] = 0.027
*wR*(*F* ^2^) = 0.074
*S* = 1.14
3770 reflections
277 parameters
H-atom parameters constrained
Δρ_max_ = 0.76 e Å^−3^
Δρ_min_ = −0.57 e Å^−3^

### 

Data collection: *CAD-4 EXPRESS* (Enraf–Nonius, 1994 ▶); cell refinement: *CAD-4 EXPRESS*; data reduction: *XCAD4* (Harms & Wocadlo, 1995 ▶); program(s) used to solve structure: *SHELXS97* (Sheldrick, 2008 ▶); program(s) used to refine structure: *SHELXL97* (Sheldrick, 2008 ▶); molecular graphics: *ORTEP-3* (Farrugia, 1997 ▶); software used to prepare material for publication: *WinGX* publication routines (Farrugia, 1999 ▶).

## Supplementary Material

Crystal structure: contains datablock(s) global, I. DOI: 10.1107/S160053681104133X/ff2028sup1.cif

Structure factors: contains datablock(s) I. DOI: 10.1107/S160053681104133X/ff2028Isup2.hkl

Additional supplementary materials:  crystallographic information; 3D view; checkCIF report

## Figures and Tables

**Table 1:** Hydrogen-bond geometry (Å, °)

*D*—H⋯*A*	*D*—H	H⋯*A*	*D*⋯*A*	*D*—H⋯*A*
N1—H1*A*⋯O1	0.89	1.91	2.785 (3)	167
N1—H1*B*⋯O6^i^	0.89	1.88	2.740 (3)	162
N1—H1*C*⋯O9^ii^	0.89	1.93	2.809 (4)	168
N2—H2*A*⋯O2	0.89	1.96	2.814 (4)	160
N2—H2*B*⋯O5^ii^	0.89	2.19	2.866 (3)	133
N2—H2*C*⋯O1^iii^	0.89	1.95	2.824 (3)	169
C3—H3⋯O1^iv^	0.93	2.56	3.339 (5)	142
C13—H13⋯O6^ii^	0.93	2.53	3.394 (4)	154
C14—H14*B*⋯O3^v^	0.97	2.56	3.410 (4)	147

Symmetry codes: (i) 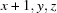; (ii) 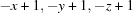; (iii) 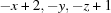; (iv) 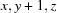; (v) 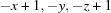.

**Fig. 1.**
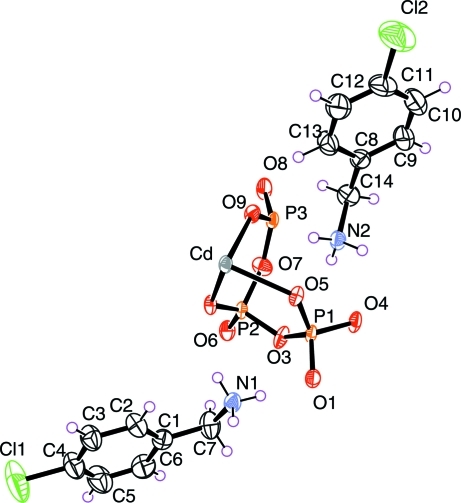
ORTEP-3 (Farrugia,(1999)) view of (I) with atom numbering scheme. Displacement ellipsoids for non-H atoms are drawn at the 30% probability level.

**Fig. 2.**
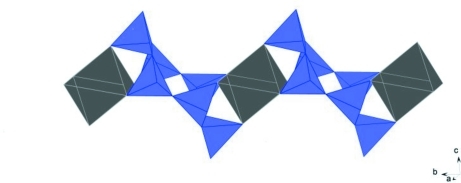
Perspective view showing the 1-D coordination polymers [Cd(P6O18)4-]n developed along the b axis.

**Fig. 3.**
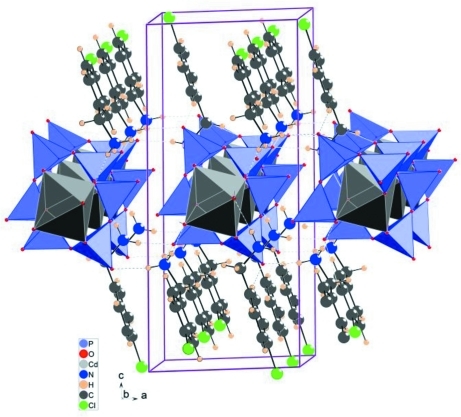
Perspective view of [Cd(P6O18)].4(CBAH) showing the the supramolecular open framework structure.

## supplementary crystallographic information

### Comment

The key to successful construction of supramolecular architecture is the control and manipulation of coordination bonds an non-covalent interactions by carefully selecting the coordination geometry of the metal atoms and the organic ligands containing appropriate functional groups (such as polyphosphoric acid and polyamine) (Kolotuchin *et al.*, 1995). Up to now, a large number of supramolecular complexes with various dimensions and topologies have been achieved through judicious choice of linkers and metal ions (Tong *et al.*, 1999). The approach to supramolecular framework employed in this work is to use the hexafunctional linker P_6_O_18_^6-^ that is of strong coordinating ability and suitable hydrogen bond acceptor. The 4-chlorobenzylamine (CBA) is used to create possibly π-π packing interactions between the aromatic rings and Cl—Cl interactions, which could facilitate the formation of ordered and non-interpenetrated open frameworks. In this contribution, we report the self-assembly of Cd^II^ with P_6_O_18_^6-^ in the presence of template (CBA) into a supramolecular open framework material [Cd(P_6_O_18_^6-^)]_n_.4n(CABH) (Scheme I). Single-crystal X-ray diffraction study of this compound shows that the asymmetric unit contains half of cadmium atom, half of a cycle P_6_O_18_ and two crystallographically independent 4-chlorobenzylammonium (CBAH) cations (Fig. 1). The Cd atom locates on an inversion centre and is coordinated by six O atoms. The CdO_6_ octahedron, sharing six vertex oxygen atoms with two adjacent P_6_O_18_ rings, is slightly distorted compared to other cases (Du *et al.*, 2010; Kontturi *et al.*, 2005; Man *et al.*, 2006; Hu *et al.*, 2008). Bond distances Cd—O range from 2.230 (2) to 2.353 (7)Å and angles O—Cd—O range from 83.53 (8) to 88.19 (7) °. The P_6_O_18_ units display average P–O distances of 1.540 A and P–P distances of 2.967 Å, values usually found in other condensed anions (Averbuch-Pouchot & Durif, 1996). The values of the P—P—P angles, varying from 87.13 (1) to 128.55 (1)°, are in the range of values observed with other cyclohexaphosphates (Durif, 2005). As shown in Fig. 2, the Cd^II^ atoms are bridged by P_6_O_18_ rings to form infinite 1-D coordination polymers [Cd(P_6_O_18_)]n parallel to the *b* axis. Fig. 3 shows the supramolecular open framework structure built of the infinite zigzag chains linked by hydrogen bonds of types N(C)—H···O ranging from 2.714 (3) to 3.410 (4) Å, established by the protonated amine (CBAH). The phenyl rings of these organic molecules are planar, with a mean plane deviation of 0.0043 Å and are parallel with a dihedral angle of 4.94°. The orientations of the –CH_2_—NH_3_^+^ substituent in the two cations (CBAH) are distinct, as seen from the following torsion angles: N1—C7—C1—C2 = 13.1 (3) and N2—C14—C8—C9 = 118.3 (17)°. The supramolecular framework structure is further stabilized by electrostatic strengths, Cl–Cl interactions [4.051 Å] (Hathwar *et al.*, 2010). The inter-planar distance between nearby phenyl rings is in the vicinity of 4.165 Å, which is longer than 3.80 Å, value required for the formation of π–π interactions (Janiak *et al.*, 2000).

### Experimental

The chemicals used to prepare the title compounds include CdCO_3_, 4-chlorobenzylamine (CBA) and H_6_P_6_O_18_. Both first reagents were commercially available (Accros), the third one was produced from Li_6_P_6_O_18_.6H_2_O, which is prepared by the process of Schülke (Schülke & Kayser, 1985) and protonated with an ion-exchange resin (Amberlite IR 120) in its H-state. An aqueous solution of H_6_P_6_O_18_ (5 mmol, 15 ml) was added dropwise to a stirred mixture of CdCO_3_ (0.86 g, 5 mmol), 4-chlorobenzylamine (2.45 ml, 20 mmol)and C_2_H_5_OH (50 ml). The obtained solution was allowed to stand in air at room temperature until formation of single crystals of the title complex.

### Refinement

All H atoms were positioned geometrically and treated as riding on their parent atoms, [N–H = 0.89 with *U*_iso_(H) = 1.5Ueq, C–H =0.96 Å (CH_3_) and C–H =0.96 Å (Ar–H), with *U*_iso_(H) = 1.2Ueq].

### Crystal data

**Table:**

(C_7_H_9_ClN)_4_[Cd(P_6_O_18_)]	*Z* = 1
*M**_r_* = 1156.63	*F*(000) = 582
Triclinic, *P*1	*D*_x_ = 1.741 Mg m^−^^3^
Hall symbol: -P 1	Mo *K*α radiation, λ = 0.71073 Å
*a* = 8.021 (4) Å	Cell parameters from 25 reflections
*b* = 8.1696 (16) Å	θ = 9.1–10.8°
*c* = 17.919 (3) Å	µ = 1.03 mm^−^^1^
α = 87.31 (5)°	*T* = 293 K
β = 88.914 (19)°	Prism, colourless
γ = 70.100 (3)°	0.22 × 0.20 × 0.18 mm
*V* = 1102.9 (6) Å^3^	

### Data collection

**Table:**

Enraf–Nonius TurboCAD-4 diffractometer	*R*_int_ = 0.009
Radiation source: Enraf Nonius FR590	θ_max_ = 28.0°, θ_min_ = 2.3°
graphite	*h* = −9→10
non–profiled ω scans	*k* = 0→10
3873 measured reflections	*l* = −5→23
3770 independent reflections	2 standard reflections every 120 min
3506 reflections with *I* > 2σ(*I*)	intensity decay: 1%

### Refinement

**Table:**

Refinement on *F*^2^	Primary atom site location: structure-invariant direct methods
Least-squares matrix: full	Secondary atom site location: difference Fourier map
*R*[*F*^2^ > 2σ(*F*^2^)] = 0.027	Hydrogen site location: inferred from neighbouring sites
*wR*(*F*^2^) = 0.074	H-atom parameters constrained
*S* = 1.14	*w* = 1/[σ^2^(*F*_o_^2^) + (0.0373*P*)^2^ + 0.863*P*] where *P* = (*F*_o_^2^ + 2*F*_c_^2^)/3
3770 reflections	(Δ/σ)_max_ = 0.022
277 parameters	Δρ_max_ = 0.76 e Å^−^^3^
0 restraints	Δρ_min_ = −0.57 e Å^−^^3^

### Special details

**Table:**

Geometry. All e.s.d.'s (except the e.s.d. in the dihedral angle between two l.s. planes) are estimated using the full covariance matrix. The cell e.s.d.'s are taken into account individually in the estimation of e.s.d.'s in distances, angles and torsion angles; correlations between e.s.d.'s in cell parameters are only used when they are defined by crystal symmetry. An approximate (isotropic) treatment of cell e.s.d.'s is used for estimating e.s.d.'s involving l.s. planes.
Refinement. Refinement of *F*^2^ against ALL reflections. The weighted *R*-factor *wR* and goodness of fit *S* are based on *F*^2^, conventional *R*-factors *R* are based on *F*, with *F* set to zero for negative *F*^2^. The threshold expression of *F*^2^ > σ(*F*^2^) is used only for calculating *R*-factors(gt) *etc*. and is not relevant to the choice of reflections for refinement. *R*-factors based on *F*^2^ are statistically about twice as large as those based on *F*, and *R*- factors based on ALL data will be even larger.

### Fractional atomic coordinates and isotropic or equivalent isotropic displacement parameters (Å^2^)

**Table:**

	*x*	*y*	*z*	*U*_iso_*/*U*_eq_	
P1	0.67291 (7)	0.07788 (7)	0.58321 (4)	0.02160 (17)	
P2	0.31753 (7)	0.30435 (7)	0.62408 (4)	0.02213 (17)	
P3	0.21259 (7)	0.28764 (8)	0.46566 (4)	0.02360 (18)	
O1	0.8257 (2)	0.0489 (2)	0.63383 (12)	0.0306 (5)	
O2	0.6552 (2)	0.1994 (2)	0.51710 (12)	0.0278 (5)	
O3	0.4957 (2)	0.1402 (2)	0.63318 (13)	0.0332 (5)	
O4	0.6588 (2)	−0.1001 (2)	0.55819 (13)	0.0321 (6)	
O5	0.3676 (2)	0.4624 (2)	0.60678 (11)	0.0265 (5)	
O6	0.2085 (2)	0.3003 (2)	0.69060 (12)	0.0316 (5)	
O7	0.2237 (3)	0.2579 (3)	0.55466 (12)	0.0354 (6)	
O8	0.0311 (2)	0.3219 (2)	0.44049 (13)	0.0390 (6)	
O9	0.3052 (2)	0.4116 (2)	0.43979 (12)	0.0295 (5)	
Cd	0.5000	0.5000	0.5000	0.02314 (9)	
N1	0.8521 (3)	0.3592 (3)	0.68090 (16)	0.0336 (6)	
H1A	0.8605	0.2565	0.6633	0.050*	
H1B	0.9603	0.3617	0.6892	0.050*	
H1C	0.7969	0.4438	0.6476	0.050*	
C1	0.7286 (4)	0.5549 (4)	0.7877 (2)	0.0373 (9)	
C2	0.7656 (4)	0.6922 (4)	0.7522 (2)	0.0452 (10)	
H2	0.8130	0.6803	0.7042	0.054*	
C3	0.7325 (5)	0.8486 (5)	0.7875 (2)	0.0500 (10)	
H3	0.7541	0.9424	0.7631	0.060*	
C4	0.6673 (5)	0.8608 (5)	0.8592 (2)	0.0564 (12)	
C5	0.6306 (6)	0.7272 (6)	0.8958 (3)	0.0661 (13)	
H5	0.5852	0.7391	0.9442	0.079*	
C6	0.6619 (5)	0.5735 (5)	0.8598 (2)	0.0522 (11)	
H6	0.6377	0.4813	0.8844	0.063*	
C7	0.7503 (5)	0.3849 (4)	0.7516 (2)	0.0502 (11)	
H7A	0.8092	0.2893	0.7867	0.060*	
H7B	0.6336	0.3799	0.7420	0.060*	
Cl1	0.6261 (2)	1.05722 (18)	0.90321 (8)	0.1103 (6)	
N2	0.8179 (3)	0.1736 (3)	0.37553 (14)	0.0286 (6)	
H2A	0.7756	0.1534	0.4200	0.043*	
H2B	0.8255	0.2800	0.3734	0.043*	
H2C	0.9251	0.0952	0.3689	0.043*	
C8	0.7590 (4)	0.1923 (4)	0.24011 (19)	0.0358 (8)	
C9	0.8004 (5)	0.0633 (5)	0.1879 (2)	0.0564 (12)	
H9	0.7943	−0.0456	0.2019	0.068*	
C10	0.8495 (7)	0.0933 (6)	0.1169 (3)	0.0694 (15)	
H10	0.8750	0.0059	0.0826	0.083*	
C11	0.8614 (6)	0.2538 (6)	0.0958 (2)	0.0589 (12)	
C12	0.8237 (5)	0.3838 (5)	0.1459 (2)	0.0558 (11)	
H12	0.8325	0.4915	0.1317	0.067*	
C13	0.7730 (4)	0.3532 (4)	0.2169 (2)	0.0453 (10)	
H13	0.7472	0.4416	0.2507	0.054*	
C14	0.6978 (4)	0.1610 (4)	0.3163 (2)	0.0401 (9)	
H14A	0.5808	0.2454	0.3244	0.048*	
H14B	0.6877	0.0460	0.3198	0.048*	
Cl2	0.9233 (3)	0.2940 (2)	0.00535 (8)	0.1069 (6)	

### Atomic displacement parameters (Å^2^)

**Table:**

	*U*^11^	*U*^22^	*U*^33^	*U*^12^	*U*^13^	*U*^23^
P1	0.0171 (2)	0.0128 (2)	0.0337 (4)	−0.0031 (2)	0.0003 (3)	−0.0040 (3)
P2	0.0190 (2)	0.0152 (3)	0.0315 (4)	−0.0047 (2)	0.0032 (3)	−0.0051 (3)
P3	0.0204 (2)	0.0144 (3)	0.0364 (4)	−0.0057 (2)	−0.0049 (3)	−0.0045 (4)
O1	0.0246 (8)	0.0235 (8)	0.0421 (13)	−0.0050 (7)	−0.0056 (8)	−0.0079 (10)
O2	0.0262 (8)	0.0182 (8)	0.0356 (13)	−0.0035 (6)	0.0047 (8)	−0.0001 (10)
O3	0.0249 (8)	0.0201 (8)	0.0471 (15)	0.0005 (7)	0.0114 (9)	0.0041 (11)
O4	0.0235 (7)	0.0152 (7)	0.0566 (16)	−0.0043 (6)	−0.0065 (9)	−0.0078 (11)
O5	0.0288 (8)	0.0161 (7)	0.0355 (12)	−0.0084 (6)	0.0038 (8)	−0.0060 (10)
O6	0.0268 (8)	0.0288 (9)	0.0387 (13)	−0.0085 (7)	0.0084 (8)	−0.0065 (11)
O7	0.0427 (10)	0.0386 (11)	0.0354 (14)	−0.0273 (9)	−0.0031 (9)	−0.0022 (12)
O8	0.0230 (8)	0.0293 (9)	0.0635 (17)	−0.0050 (7)	−0.0104 (9)	−0.0154 (12)
O9	0.0334 (8)	0.0195 (8)	0.0392 (13)	−0.0133 (7)	−0.0079 (9)	−0.0007 (10)
Cd	0.02384 (11)	0.01532 (11)	0.03217 (18)	−0.00887 (9)	−0.00109 (10)	−0.00258 (13)
N1	0.0303 (10)	0.0249 (10)	0.0464 (17)	−0.0101 (9)	−0.0057 (11)	−0.0041 (13)
C1	0.0316 (12)	0.0382 (15)	0.043 (2)	−0.0126 (12)	−0.0013 (13)	−0.0018 (19)
C2	0.0535 (17)	0.0464 (18)	0.042 (2)	−0.0242 (15)	0.0066 (16)	−0.012 (2)
C3	0.068 (2)	0.0419 (17)	0.047 (3)	−0.0273 (17)	0.0068 (18)	−0.006 (2)
C4	0.074 (2)	0.050 (2)	0.049 (3)	−0.0237 (19)	0.009 (2)	−0.015 (2)
C5	0.091 (3)	0.070 (3)	0.042 (3)	−0.032 (2)	0.017 (2)	−0.012 (3)
C6	0.066 (2)	0.049 (2)	0.044 (3)	−0.0245 (18)	0.006 (2)	0.008 (2)
C7	0.0545 (18)	0.0404 (17)	0.062 (3)	−0.0249 (15)	0.0130 (18)	−0.008 (2)
Cl1	0.1789 (15)	0.0753 (8)	0.0887 (11)	−0.0554 (9)	0.0468 (10)	−0.0485 (9)
N2	0.0239 (9)	0.0246 (10)	0.0353 (15)	−0.0048 (8)	0.0001 (9)	−0.0065 (12)
C8	0.0382 (13)	0.0338 (14)	0.0370 (19)	−0.0138 (12)	−0.0073 (13)	−0.0016 (17)
C9	0.081 (2)	0.0355 (17)	0.053 (3)	−0.0185 (17)	−0.008 (2)	−0.010 (2)
C10	0.112 (3)	0.048 (2)	0.045 (3)	−0.022 (2)	0.002 (2)	−0.015 (3)
C11	0.082 (3)	0.064 (2)	0.030 (2)	−0.025 (2)	0.0006 (19)	0.003 (3)
C12	0.072 (2)	0.0465 (19)	0.054 (3)	−0.0269 (18)	−0.006 (2)	0.000 (2)
C13	0.0578 (18)	0.0387 (17)	0.045 (2)	−0.0227 (15)	0.0010 (16)	−0.009 (2)
C14	0.0350 (13)	0.0433 (16)	0.048 (2)	−0.0211 (12)	−0.0060 (14)	−0.0035 (19)
Cl2	0.1687 (16)	0.1080 (12)	0.0444 (8)	−0.0486 (12)	0.0145 (9)	−0.0017 (10)

### Geometric parameters (Å, °)

**Table:**

P1—O1	1.4846 (19)	C3—C4	1.372 (6)
P1—O2	1.486 (2)	C3—H3	0.9300
P1—O4	1.5822 (16)	C4—C5	1.362 (6)
P1—O3	1.607 (2)	C4—Cl1	1.748 (3)
P2—O6	1.471 (2)	C5—C6	1.383 (5)
P2—O5	1.4947 (18)	C5—H5	0.9300
P2—O7	1.5909 (19)	C6—H6	0.9300
P2—O3	1.5975 (18)	C7—H7A	0.9700
P3—O8	1.4614 (18)	C7—H7B	0.9700
P3—O9	1.499 (2)	N2—C14	1.478 (3)
P3—O4^i^	1.6009 (16)	N2—H2A	0.8900
P3—O7	1.601 (2)	N2—H2B	0.8900
O2—Cd	2.3534 (18)	N2—H2C	0.8900
O4—P3^i^	1.6009 (16)	C8—C9	1.392 (4)
O5—Cd	2.230 (2)	C8—C13	1.401 (5)
O9—Cd	2.2432 (17)	C8—C14	1.482 (5)
Cd—O5^ii^	2.230 (2)	C9—C10	1.361 (6)
Cd—O9^ii^	2.2432 (17)	C9—H9	0.9300
Cd—O2^ii^	2.3534 (18)	C10—C11	1.382 (7)
N1—C7	1.478 (4)	C10—H10	0.9300
N1—H1A	0.8900	C11—C12	1.374 (5)
N1—H1B	0.8900	C11—Cl2	1.735 (5)
N1—H1C	0.8900	C12—C13	1.365 (5)
C1—C2	1.380 (5)	C12—H12	0.9300
C1—C6	1.383 (5)	C13—H13	0.9300
C1—C7	1.515 (4)	C14—H14A	0.9700
C2—C3	1.393 (4)	C14—H14B	0.9700
C2—H2	0.9300		
			
O1—P1—O2	117.93 (12)	C3—C2—H2	119.7
O1—P1—O4	111.66 (10)	C4—C3—C2	118.4 (4)
O2—P1—O4	109.88 (11)	C4—C3—H3	120.8
O1—P1—O3	107.43 (12)	C2—C3—H3	120.8
O2—P1—O3	109.96 (11)	C5—C4—C3	122.2 (3)
O4—P1—O3	98.11 (10)	C5—C4—Cl1	119.5 (3)
O6—P2—O5	118.97 (11)	C3—C4—Cl1	118.2 (3)
O6—P2—O7	107.58 (11)	C4—C5—C6	118.8 (4)
O5—P2—O7	111.28 (13)	C4—C5—H5	120.6
O6—P2—O3	106.68 (13)	C6—C5—H5	120.6
O5—P2—O3	108.08 (11)	C5—C6—C1	121.0 (4)
O7—P2—O3	102.99 (12)	C5—C6—H6	119.5
O8—P3—O9	118.36 (13)	C1—C6—H6	119.5
O8—P3—O4^i^	111.32 (9)	N1—C7—C1	114.6 (3)
O9—P3—O4^i^	104.99 (10)	N1—C7—H7A	108.6
O8—P3—O7	110.18 (13)	C1—C7—H7A	108.6
O9—P3—O7	110.64 (11)	N1—C7—H7B	108.6
O4^i^—P3—O7	99.62 (12)	C1—C7—H7B	108.6
P1—O2—Cd	131.47 (10)	H7A—C7—H7B	107.6
P2—O3—P1	131.59 (16)	C14—N2—H2A	109.5
P1—O4—P3^i^	138.76 (11)	C14—N2—H2B	109.5
P2—O5—Cd	122.36 (10)	H2A—N2—H2B	109.5
P2—O7—P3	139.86 (14)	C14—N2—H2C	109.5
P3—O9—Cd	129.75 (13)	H2A—N2—H2C	109.5
O5—Cd—O5^ii^	180.0	H2B—N2—H2C	109.5
O5—Cd—O9	88.19 (7)	C9—C8—C13	117.2 (4)
O5^ii^—Cd—O9	91.81 (7)	C9—C8—C14	121.0 (3)
O5—Cd—O9^ii^	91.81 (7)	C13—C8—C14	121.8 (3)
O5^ii^—Cd—O9^ii^	88.19 (7)	C10—C9—C8	121.4 (4)
O9—Cd—O9^ii^	180.00 (10)	C10—C9—H9	119.3
O5—Cd—O2	83.53 (8)	C8—C9—H9	119.3
O5^ii^—Cd—O2	96.47 (8)	C9—C10—C11	119.8 (3)
O9—Cd—O2	83.70 (7)	C9—C10—H10	120.1
O9^ii^—Cd—O2	96.30 (7)	C11—C10—H10	120.1
O5—Cd—O2^ii^	96.47 (8)	C12—C11—C10	120.5 (4)
O5^ii^—Cd—O2^ii^	83.53 (8)	C12—C11—Cl2	119.2 (4)
O9—Cd—O2^ii^	96.30 (7)	C10—C11—Cl2	120.3 (3)
O9^ii^—Cd—O2^ii^	83.70 (7)	C13—C12—C11	119.3 (4)
O2—Cd—O2^ii^	180.000 (1)	C13—C12—H12	120.3
C7—N1—H1A	109.5	C11—C12—H12	120.3
C7—N1—H1B	109.5	C12—C13—C8	121.7 (3)
H1A—N1—H1B	109.5	C12—C13—H13	119.2
C7—N1—H1C	109.5	C8—C13—H13	119.2
H1A—N1—H1C	109.5	N2—C14—C8	113.1 (2)
H1B—N1—H1C	109.5	N2—C14—H14A	109.0
C2—C1—C6	118.9 (3)	C8—C14—H14A	109.0
C2—C1—C7	123.9 (3)	N2—C14—H14B	109.0
C6—C1—C7	117.2 (3)	C8—C14—H14B	109.0
C1—C2—C3	120.7 (3)	H14A—C14—H14B	107.8
C1—C2—H2	119.7		

Symmetry codes: (i) −*x*+1, −*y*, −*z*+1; (ii) −*x*+1, −*y*+1, −*z*+1.

### Hydrogen-bond geometry (Å, °)

**Table:**

*D*—H···*A*	*D*—H	H···*A*	*D*···*A*	*D*—H···*A*
N1—H1A···O1	0.89	1.91	2.785 (3)	167
N1—H1B···O6^iii^	0.89	1.88	2.740 (3)	162
N1—H1C···O9^ii^	0.89	1.93	2.809 (4)	168
N2—H2A···O2	0.89	1.96	2.814 (4)	160
N2—H2B···O5^ii^	0.89	2.19	2.866 (3)	133
N2—H2C···O1^iv^	0.89	1.95	2.824 (3)	169
C3—H3···O1^v^	0.93	2.56	3.339 (5)	142
C13—H13···O6^ii^	0.93	2.53	3.394 (4)	154
C14—H14B···O3^i^	0.97	2.56	3.410 (4)	147

Symmetry codes: (iii) *x*+1, *y*, *z*; (ii) −*x*+1, −*y*+1, −*z*+1; (iv) −*x*+2, −*y*, −*z*+1; (v) *x*, *y*+1, *z*; (i) −*x*+1, −*y*, −*z*+1.
